# Single‐marker and haplotype‐based genome‐wide association studies for the number of teats in two heavy pig breeds

**DOI:** 10.1111/age.13095

**Published:** 2021-06-06

**Authors:** S. Bovo, M. Ballan, G. Schiavo, A. Ribani, S. Tinarelli, V. J. Utzeri, S. Dall'Olio, M. Gallo, L. Fontanesi

**Affiliations:** ^1^ Department of Agricultural and Food Sciences Division of Animal Sciences University of Bologna Viale Fanin 46 Bologna 40127 Italy; ^2^ Associazione Nazionale Allevatori Suini (ANAS) Via Nizza 53 Roma 00198 Italy

**Keywords:** *ARL4C*, *FRMD4A*, *HOXB1*, Landrace, Large White, morphological trait, single nucleotide polymorphism, *Sus scrofa*, *VRTN*

## Abstract

The number of teats is a reproductive‐related trait of great economic relevance as it affects the mothering ability of the sows and thus the number of properly weaned piglets. Moreover, genetic improvement of this trait is fundamental to parallelly help the selection for increased litter size. We present the results of single‐marker and haplotypes‐based genome‐wide association studies for the number of teats in two large cohorts of heavy pig breeds (Italian Large White and Italian Landrace) including 3990 animals genotyped with the 70K GGP Porcine BeadChip and other 1927 animals genotyped with the Illumina PorcineSNP60 BeadChip. In the Italian Large White population, genome scans identified three genome regions (SSC7, SSC10, and SSC12) that confirmed the involvement of the *VRTN* gene (as we previously reported) and highlighted additional loci known to affect teat counts, including the *FRMD4A* and *HOXB1* gene regions. A different picture emerged in the Italian Landrace population, with a total of 12 genome regions in eight chromosomes (SSC3, SSC6, SSC8, SSC11, SSC13, SSC14, SSC15, and SSC16) mainly detected via the haplotype‐based genome scan. The most relevant QTL was close to the *ARL4C* gene on SSC15. Markers in the *VRTN* gene region were not significant in the Italian Landrace breed. The use of both single‐marker and haplotype‐based genome‐wide association analyses can be helpful to exploit and dissect the genome of the pigs of different populations. Overall, the obtained results supported the polygenic nature of the investigated trait and better elucidated its genetic architecture in Italian heavy pigs.

## Introduction

The number of piglets weaned per sow and per year is one of the most important indicators of economic sustainability of pig farming. This is a multifactorial and complex parameter that is affected, in part, by the mothering ability of the sows, which in turn, is related to the number of their teats that have to supply the needed nutrients to all piglets (Kim et al. 2005; Andersen et al. 2011). Therefore, an adequate number of teats is required to parallelly help the selection for increased litter size and that, together, can maximise the number of weaned piglets.

The number of teats in pigs is considered a quantitative trait (but with discrete and countable values) that shows a considerable variability among and within breeds and lines (Borchers et al. 2002; Lopes et al. 2014; Verardo et al. 2016; Rohrer & Nonneman 2017; Dall'Olio et al. 2018; van Son et al. 2019). The medium/high level of heritability of this parameter can facilitate selection to improve overall sow reproductive performances (e.g. Willham & Whatley 1962; Toro et al. 1986; McKay & Rahnefeld 1990; Borchers et al. 2002; Chalkias et al. 2013; Felleki & Lundeheim 2015; Balzani et al. 2016).

Several studies have identified quantitative trait loci (QTL) for the number of teats by using reference populations constituted by crossing pigs of different breeds or lines, including some highly hyper‐prolific Chinese breeds (e.g. Wada et al. 2000; Hirooka et al. 2001; Rodríguez et al. 2005; Bidanel et al. 2008; Ding et al. 2009; Hernandez et al. 2014) or by applying genome‐wide association studies (GWAS) within breeds (e.g. Arakawa et al. 2015; Rohrer & Nonneman 2017; Tang et al. 2017; Lee et al. 2019; van Son et al. 2019). Results of these studies evidenced many different QTL affecting this trait and confirmed its polygenic nature. Porcine chromosome (SSC) 7 has been reported to harbor one of the most important QTL affecting this trait segregating in several populations (Mikawa et al. 2007, 2011; Duijvesteijn et al. 2014; Rohrer & Nonneman 2017; Dall'Olio et al. 2018; van Son et al. 2019; Moscatelli et al. 2020). This QTL has pleiotropic effects on the number of vertebrae due to variability in the *vertebrae development associate*d gene known also as *vertnin* (*VRTN*) (Mikawa et al. 2011; Arakawa et al. 2015), which encodes for a DNA binding factor (Duan et al. 2018). A few other studies proposed that polymorphic sites close to the *VRTN* gene and variability in other genes located on SSC7 (*latent transforming growth factor binding protein 2* or *LTBP2* and *BRMS1‐like transcriptional repressor* or *BRMS1L*) could affect the number of teats and vertebrae in pigs (Zhang et al. 2016; Park et al. 2018).

GWAS may benefit from utilising haplotypes instead of SNPs to establish marker‐phenotype associations (Lorenz et al. 2010; Barendse 2011). We recently demonstrated that by using haplotypes in GWAS it is possible to detect genomic regions affecting targeted phenotypes that could not be detected with a single‐marker approach and vice versa (Bovo et al. 2019, 2020a). The additional information that could be extracted might be dependent on the particular mutational and recombination history between QTL alleles and the genotyped markers, which in turn, might be affected by possible ascertainment biases derived by the construction of the SNP genotyping panels used in the studies. Therefore, the use of both approaches has been recommended to take advantage of the full information content of the genotype data (Lorenz et al. 2010; Barendse 2011).

In our previous studies, we described the variability of the number of teats in the Italian Large White pig breed and evaluated the effect of DNA polymorphisms (in candidate genes and from single‐marker genome‐wide analyses) on this phenotype (Dall'Olio et al. 2018; Moscatelli et al. 2020).

In this study, we carried out GWAS in a much larger cohort of Italian Large White pigs and in the Italian Landrace breed (not previously investigated) that made it possible to identify additional candidate gene regions associated with the number of teats. Genomic analyses were first based on linear mixed models fitting single SNPs and then on haplotypes, that further refined the results and identified additional loci associated with this important trait.

## Materials and Methods

### Animals

All animals included in this study were from the national selection programme of heavy pig breeds that is run by the Italian Pig Breeders Association (ANAS). Pigs investigated were 3974 of the Italian Large White (866 castrated males and 1688 gilts and 1420 sows) and of 1943 Italian Landrace (106 castrated males and 263 gilts and 1574 sows) breeds. Castrated males and gilts were from the sib‐testing programme and sows were from the female selection programme. Italian Large White pigs were born in the 1985–2018 whereas Italian Landrace pigs were born in the 2006–2018.

The number of teats on these animals was routinely recorded by direct counting at the beginning of the performance testing period for the pigs included in the sib‐testing programme and at the end of the puberty for the sows that were not performance tested. Pigs having fewer than 14 teats are then discarded from the herd books of the two breeds. However, in this study, to maximise variability for this trait we included a few animals with a lower number of teats for which blood could be available in the ANAS biobank.

### Genotyping and SNP quality

Blood was collected routinely from all pigs included in the national selection programme. DNA was extracted from these samples using the Wizard Genomic DNA Purification kit (Promega Corporation, Madison, WI, USA). A total of 1943 Italian Landrace and 2047 Italian Large White pigs were genotyped with the 70K GGP Porcine BeadChip (GeneSeek, Lincoln, NE), which interrogates 68516 SNPs. The remaining 1927 Italian Large White pigs were genotyped with the Illumina PorcineSNP60 BeadChip v.2 (which interrogates 61565 SNPs). Standard genotyping protocols, based on the supplier’s recommendations, were used. PLINK v.1.09 (Chang et al. 2015) was used to discard SNPs presenting a call rate <0.95, a minor allele frequency (MAF) < 0.01 and that were not in Hardy–Weinberg equilibrium (*P* < 0.001). SNPs shared between the two genotyping platforms were used in the analysis of the Italian Large White pigs. BLAST+ v.2.7.1 (Camacho et al. 2009) was used to map SNPs to the Sscrofa11.1 reference genome and markers assigned to more than one position or assigned to sex chromosomes were discarded. The genomic dataset was supplemented with the genotype status of the *VRTN* g.20311_20312ins291 mutation analyzed in 778 Italian Landrace pigs already genotyped with 70K GGP Porcine BeadChip. The genotyping protocol of this mutation was as previously described (Fontanesi et al. 2014).

### Phasing and haplotype detection

Genotypes were phased using SHAPEIT v.2 (Delaneau et al. 2011) considering a window size of 2 Mb, an effective population size (*N_e_*) estimated with SNeP v.1.1 (Barbato et al. 2015) and a chromosome specific recombination rate given by Tortereau et al. (2012). Haplotypes were further called with the R package GHap 1.2.2. Following the study by Veroneze et al. (2013), a genome window of 400 kb with a sliding block of 100 kb was used to call haplotypes. Haplotypes were exported in the *tped* file format, where haplotype allele counts 0, 1, and 2 are recoded as NN, NH, and HH genotypes (H = haplotype allele and N = NULL = all other N alleles), as if haplotypes were bi‐allelic markers. A regular ped file was obtained with PLINK, filtering out haplotypes presenting a MAF < 0.01.

### Genome‐wide association analyses

After filtering, the Italian Large White dataset counted 3888 animals (106 castrated males, 262 gilts and 1573 sows) for which 36,243 SNPs and 196,394 haplotypes (corresponding to 21231 haploblocks) were analysed. The Italian Landrace dataset was based on 1941 animals (106 castrated males, 262 gilts and 1573 sows), 50,453 SNPs and 237087 haplotypes (corresponding to 21,707 haploblocks). Breed specific GWAS were based either on SNPs or on haplotypes. Table S1 summarises the datasets used in the GWAS in the two breeds.

Association analyses were performed using an additive genetic model assuming a trend per copy of the minor allele that specify the dependency of the number of teats on genotype categories. The following linear mixed effect model was specified:

(1) y=Wα+xβ+g+e

where ***y*** (*n* × 1) is a vector containing the phenotype (the number of teats) for the *n* ^th^ animal, ***W*** (*n* × *k*) is a covariate matrix with *k* = 2 (a column of 1s and sex) and ***α*** is the *k*‐dimensional vector of covariates effects, ***x*** (*n* × 1) is the vector containing genotypes for the *i* ^th^ DNA marker (SNP or haplotype), *β* is the additive fixed effect of the *i* ^th^ DNA marker on the phenotype, ***g*** ~ N(**0**,*σ* ^2^_g_ **K**) is a multivariate Gaussian polygenic effect, with covariance matrix proportional to the relatedness matrix **K** (*n* × *n*) and **e** ~ N(**0**,*σ* ^2^_e_ **I**) is a multivariate Gaussian vector of uncorrelated residuals. The assessment of the association between each DNA marker and the total number of teats was obtained by testing the null hypothesis H_0_:β = 0. Significance was tested by using the Wald test. All the models were fitted with GEMMA v.0.98 (Zhou & Stephens 2012) after computing the relatedness matrices **K_1_** and **K_2_** as centred genomic matrices, for SNPs and haplotypes respectively. To account for multiple comparisons, we opted for the Bonferroni correction, which considered the total number of DNA markers or haplotypes and a value of *α* = 0.05. SNPs and haplotypes presenting the lowest *P* in chromosome regions separated by at least 5 Mb were considered as tag DNA markers. For each trait, GEMMA was used to estimate the genomic (chip) heritability ($h_{G}^{2}$). Genomic control inflation factors (λ_GC_), defined as the median of the resulting chi‐squared test statistics divided by the expected median of the chi‐squared distribution, were computed in R v.3.6.0 (R Core Team 2018). Quantile–quantile plotslots) and Manhattan plots were generated in R by using the *qqman* package (Turner 2018).

### Haploblocks and annotation of associated SNPs and haplotypes

QTL regions were analysed using HaploView v.4.2 (https://www.broadinstitute.org/haploview/haploview) to compare haploblock structures between the two pig breeds. Only markers shared by the two datasets were retained. Protein coding genes annotated in the Sscrofa11.1 genome version spanning the significantly associated haplotypes and a region of ± 500 kb around the significantly associated SNPs were retrieved from the Sscrofa11.1 NCBI's GFF file by using Bedtools v.2.17.0 (Quinlan & Hall 2010). Comparative QTL mapping analysis across studies was obtained using the Pig QTL database (Pig QTLdb; release 39; Hu et al. 2019). QTLs were downloaded, checked and manually curated as previously reported (Bovo et al. 2020c). The final dataset comprised a total of 295 traits and 1978 QTL regions.

## Results

### Descriptive statistics on the number of teats in the two heavy pig breeds

**Figure 1:**
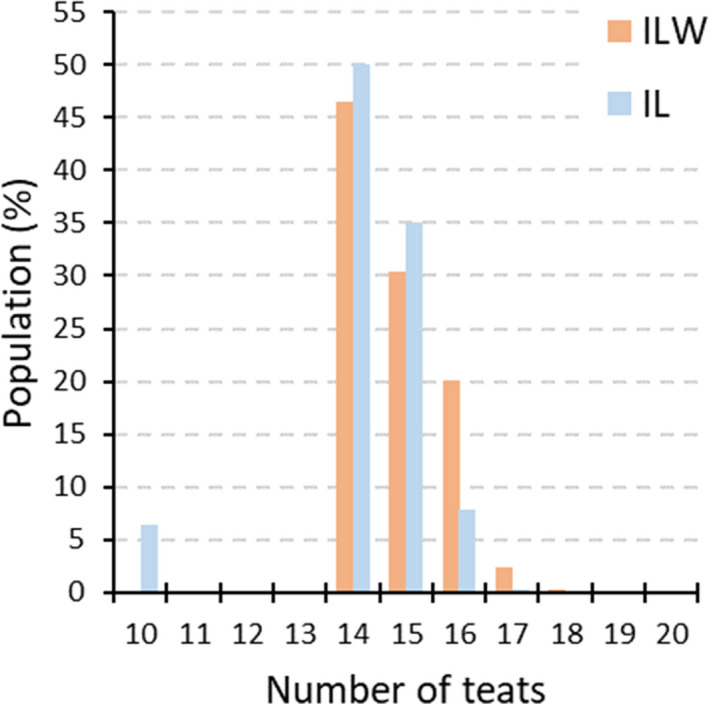
Distribution of the number of teats within the Italian Large White (ILW) and Italian Landrace (IL) populations.

Descriptive statistics on the number of teats in Italian Large White and Italian Landrace breeds are reported in Tables S2 and S3 respectively. The number of teats ranged from 12 to 20 and from 10 to 18, for the Italian Large White and Italian Landrace pigs respectively. About 46.5% (Italian Large White) and 50.1% (Italian Landrace) of the investigated pigs had 14 teats (Fig. 1), which is the lower limit considered for the registration of the animals to the Herd Book of these Italian heavy pig breeds. The percentage of pigs with more than 14 teats was 53.4% and 43.5% respectively. Within the Italian Large White population, a statistically significant difference in teat counts (*P* = 0.002, Wilcoxon rank sum test) was observed between males and females: males had on average 14.88 teats (SD = 0.92; median = 15), whereas females had on average 14.77 teats (SD = 0.87; median = 15).

Genomic heritability of the number of teats, estimated using SNP and haplotype data, was 0.25 (SE = 0.02) and 0.31 (SE = 0.03) in the Italian Large White breed and 0.30 (SE = 0.03) and 0.43 (SE = 0.04) in the Italian Landrace pigs.

### Single‐marker and haplotype‐based genome‐wide analyses in the two heavy pig breeds

**Figure 2:**
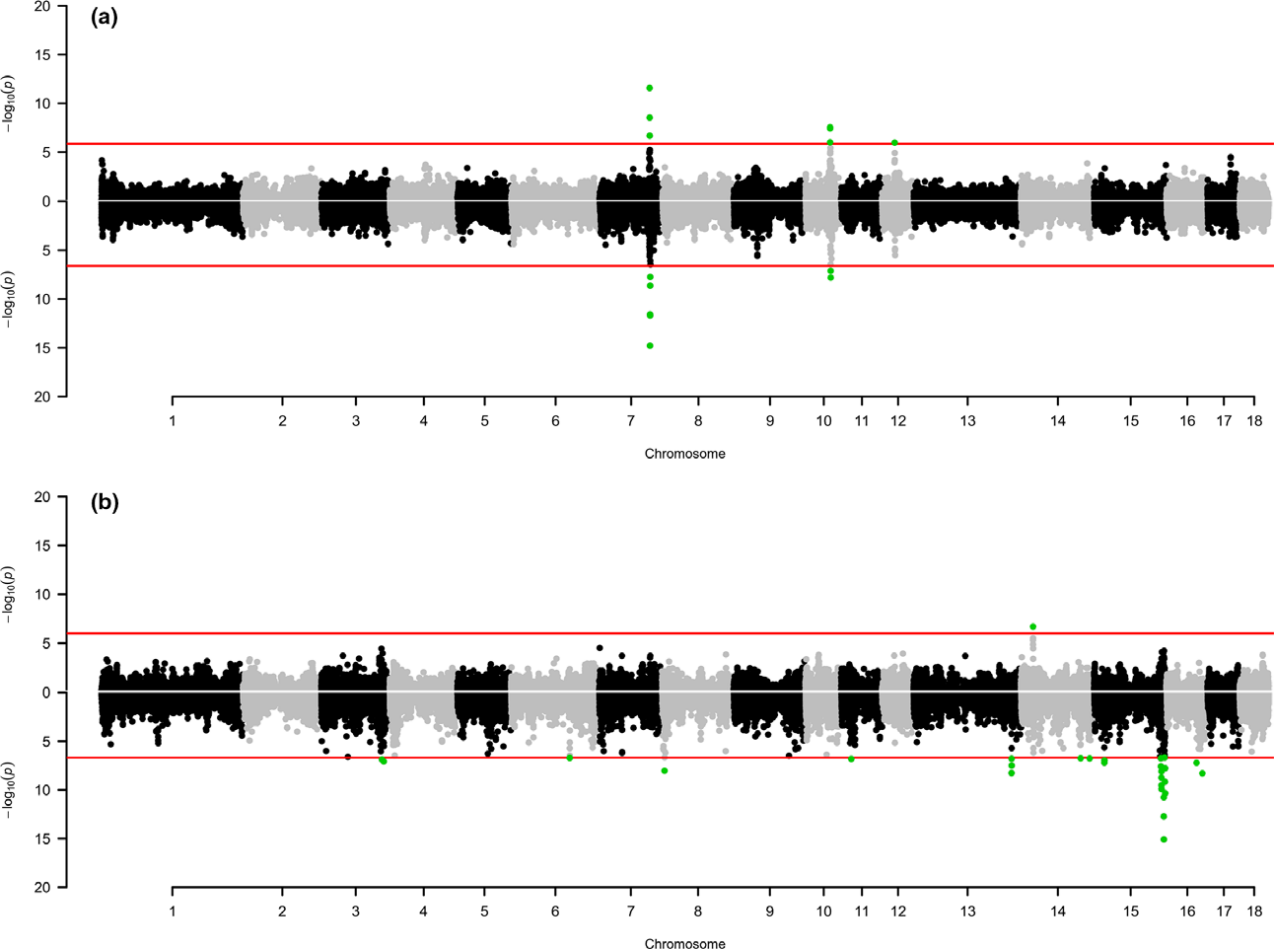
Manhattan plots of the genome‐wide association studies on the number of teats in the Italian Large White (a) and in the Italian Landrace (b) populations. Single‐marker analysis is on the top part of each plot and haplotype‐based analysis is on the bottom part of each plot. Each dot represents a DNA marker or a haplotype. The red lines identify the significance thresholds (Bonferroni correction; *α* = 0.05).

Results of the genome‐wide association studies are summarised in Table 1 that reports the top associated DNA markers (SNPs and haplotypes) for each genomic region. This table also includes information on QTLs for teat count, vertebrae count, and reproductive traits reported in the Pig QTLdb that overlapped the significant markers/regions detected in our study. Table S4 reports the full set of associated DNA markers (SNPs and haplotypes). Fig. 2 shows the Manhattan plots for the number of teats in the Italian Large White and Italian Landrace pig populations. Quantile–quantile plots are available as Fig. S1. Box plots showing the effect of the haplotype/SNP alleles at all identified QTLs are included in Figs S2 and S3 for the Italian Large White and the Italian Landrace breeds, respectively.

**Table 1:** Top genome regions associated with the number of teats in Italian Large White and Italian Landrace pigs. Results are stratified by population and chromosome.

Genome scan^1^	SSC^2^	Pos^3^	Marker^4^	Min/Maj^5^	MAF^6^	*β* ^7^	*P* ^8^	QTLs^9^	Genes^10^
Italian Large White									
Haplotype‐based	7	97435001	CHR7_B973	H/N	0.426	0.188	1.61 × 10^−15^	NNF, TVN, VN	*ENTPD5, FAM161B*, ***VRTN****, ABCD4, ALDH6A1, SYNDIG1L, VSX2, BBOF1, COQ6, LIN52, ZNF410*
Single‐marker	7	97652632	MARC0038565	A/G	0.482	−0.163	2.65 × 10^−12^	TVN	***VRTN*** *, SYNDI1GL*
Single‐marker	10	47751164	M1GA0014145	A/G	0.477	−0.135	2.83 × 10^−8^	–	***FRMD4A***
Haplotype‐based	10	48035001	CHR10_B479	H/N	0.367	−0.129	1.56 × 10^−8^	LTN	***FRMD4A*** *, PRPF18, BEND7*
Single‐marker	12	24723142	MARC0031045	G/A	0.358	0.117	1.03 × 10^−6^	THVN	*SKAP1*, ***HOXB1***
Italian Landrace									
Haplotype‐based	3	120600001	CHR3_B1205	H/N	0.014	−1.048	8.40 × 10^−8^	THVN	*RAD51AP2, FAM49A*
Haplotype‐based	6	113000001	CHR6_B1129	H/N	0.062	−0.466	1.95 × 10^−7^	–	*CDH2*
Haplotype‐based	8	4500001	CHR8_B44	H/N	0.011	−1.142	9.30 × 10^−9^	NFN	*C8H4orf50, JAKMIP1, WFS1, LOC102165510, PPP2R2C*
Haplotype‐based	11	18900001	CHR11_B188	H/N	0.034	−0.529	1.55 × 10^−7^	–	*FNDC3A, CYSLTR2, RCBTB2*
Haplotype‐based	13	189100001	CHR13_B1890	H/N	0.016	−0.957	5.43 × 10^−9^	MI	*ATP5J, GABPA, JAM2, MRPL39*
Haplotype‐based	14	132300001	CHR14_B1322	H/N	0.014	−0.953	1.73 × 10^−7^	–	*C14H10orf120, LOC100620521, AWN, LOC100519221, FAM24B, SPMI, DMBT1, PSP‐II, AQN‐1, HTRA1, PSP‐I*
Haplotype‐based	14	114800001	CHR14_B1147	H/N	0.051	−0.547	1.79 × 10^−7^	–	*COL17A1, SH3PXD2A, STN1, SLK*
Single‐marker	14	23582019	WU_10.2_14_25047530	A/G	0.078	−0.445	3.18 × 10^−7^	–	*SFSWAP*
Haplotype‐based	15	134400001	CHR15_B1343	H/N	0.030	−0.951	8.08 × 10^−16^	BW, BL, LW, TNBA, LS	*TRPM8, SPP2* Close to ***ARL4C***
Haplotype‐based	15	19300001	CHR15_B192	H/N	0.013	−0.877	6.29 × 10^−8^	–	*NCKAP5*
Haplotype‐based	16	57400001	CHR16_B573	H/N	0.024	−0.806	6.15 × 10^−8^	–	*TENM2*
Haplotype‐based	16	68700001	CHR16_B686	H/N	0.017	−0.993	5.08 × 10^−9^	–	*SAP30L, HAND1, GALNT10*

^1^ Genome scans performed within each pig population. SNPs and Haplotypes indicate which DNA markers have been used to carry out the genome scans.

^2^ *Sus scrofa* chromosome.

^3^ Position, in bp, on the *Sus scrofa* reference genome (v.11.1).

^4^ DNA marker identifier reported in the chip panels. For haplotypes, the haploblock identifier (chromosome specific) is reported.

^5^ Minor/Major alleles. Haplotypes have been treated as bi‐allelic variants (H = haplotype allele and N = other N alleles).

^6^ Minor allele frequency.

^7^ Regression coefficient. A positive value indicates that the no. of teats increases with the increasing of the number of copies of the minor allele. A negative value indicates that the no. of teats decreases with the increasing of the number of copies of the minor allele.

^8^ *P* at the Wald test (GEMMA).

^9^ QTLs related to reproductive traits overlapping the association peaks. Short names for QTL are: BL, body length; BW, body width; LS, litter size; LTN, left teat number; LWT, litter weight; MI, maternal infanticide; NFN, non‐functional nipples; NNF, number of non‐viable foetuses; THVN, thoracolumbar vertebra number; TNBA, total number of born alive; TVN, thoracic vertebra number; VN, vertebra number.

^10^ Genes overlapping the haplotype or closed to a SNP. The candidate genes are reported in bold.

**Figure 3:**
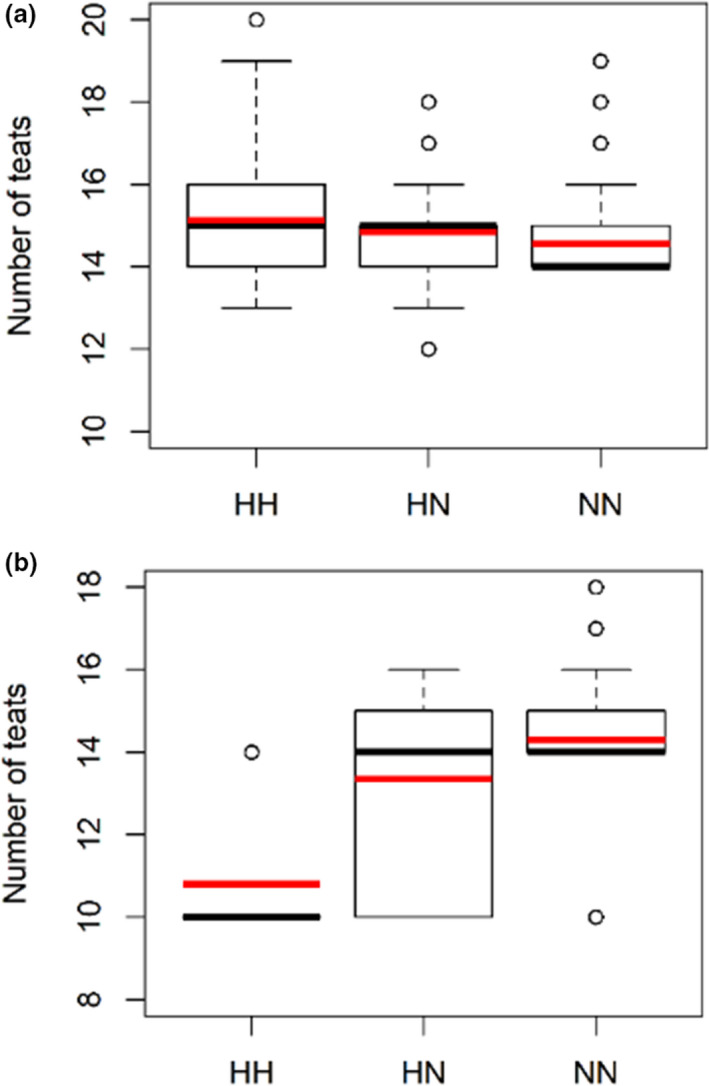
Boxplots showing the allelic effects of the top associated haplotype regions for the number of teats. (a) Genome region SSC7:97435001 bp in Italian Large White pigs and (b) genome region SSC15:134400001 bp in Italian Landrace pigs. Haplotypes have been treated as bi‐allelic variants (H = haplotype allele and N = other N alleles). The red line indicates the average value.

*Genome scans in the Italian Large White breed* – Three genomic regions in three different chromosomes (SSC7, SSC10, and SSC12) were associated with the analysed trait in this breed (Table 1 and Fig. 2a). The most significant SNP was MARC0038565 (*P* = 2.65 × 10^−12^), located on SSC7 at position 97 652 632 bp, in the region of the *VRTN* gene. This peak was also detected by the haplotype analysis (*P* = 1.61 × 10^−15^), confirming the involvement of the *VRTN* gene region in affecting the number of teats in the Italian Large White pig population (Dall'Olio et al. 2018; Moscatelli et al. 2020). Fig. 3a shows the allelic effects of this top associated haplotype region. Genome scans confirmed the direction of the effects of the MARC0038565 SNP alleles already reported by Moscatelli et al. (2020) and of the haplotypes including the alleles of this SNP.

The second major peak was observed on SSC10, in the region of the *FERM domain containing 4A* (*FRMD4A*) gene (position 47 385 718‐48 049 718 bp), with the most significant SNP (M1GA0014145; *P* = 2.83 × 10^−8^) located at position 47 751 164 bp. Haplotype analysis (*P* = 1.56 × 10^−8^) confirmed the results obtained in the single‐marker analysis and further supported the role of the *FRMD4* gene region in affecting the variability of the number of teats in this pig breed.

A third region affecting the investigated trait was detected on SSC12 with the single‐marker analysis. The significant marker MARC0031045 (*P* = 1.03 × 10^−6^), located at position 24 723 142 bp, is positioned near a cluster of Hox genes, including the *homeobox* *B1* (*HOXB1*) gene (position 24 491 486–24 492 906 bp). According to the key role of this gene (and other genes of the same family) in the developmental regulatory system, *HOXB1* (and/or other Hox genes) could be considered the candidate gene(s) of this region. For comparative analysis between breeds, Table S5 reports the effect of the top associated SNPs when they were investigated in Italian Landrace pigs.

*Genome scans in the Italian Landrace breed* – A total of 12 genomic regions (11 detected with the haplotype‐based analysis and one with the single‐marker analysis) in eight different chromosomes (SSC3, SSC6, SSC8, SSC11, SSC13, SSC14, SSC15, and SSC16) were associated with the number of teats in this breed (Table 1 and Fig. 2). Haplotype or SNP alleles determining a reduced number of teats were the less frequent in the population (Table 1, Fig. S3). Among all these QTL regions, only five were reported to be located in chromosome regions in which other studies already reported QTL directly or indirectly related to teat number (Table 1). The most significant region, detected with the haplotype‐based analysis (*P* = 8.1 × 10^−16^), was on SSC15 (positions 134 200 001–134 600 001 bp). This is the only region detected in the Italian Landrace breed that overlaps with a QTL region reported in a previous study to affect teat numbers (van Son et al. 2019). Fig. 3b shows the allelic effects of this top associated haplotype region. Other related QTL were already reported in this region (Table 1).

The second and third most significantly associated haplotypes were located on SSC16 (position 68.7 Mb; *P* = 5.08 × 10^−9^) and on SSC13 (position 189.1 Mb; *P* = 5.43 × 10^−9^). Only the second region was previously reported to overlap a QTL related to maternal behaviour (Table 1).

The single‐marker analysis identified a significant peak on SSC14 (position 23 582 019 bp; marker WU_10.2_14_25047530), located near the *adhesion G protein‐coupled receptor D1* (*ADGRD1*) gene (also known as *GPR133*) and proposed to be a gene associated with adult height (Tönjes et al. 2009).

### Comparative analysis of the *VRTN* genome region in the two heavy pig breeds

In the Italian Landrace breed, no significant markers or haplotypes were identified on SSC7 in the region of the *VRTN* gene. The most significant marker in this region was a SNP at position 98 763 633 bp (WU_10.2_7_104470681; *P* = 3.04 × 10^−4^) that is far away (1.11 Mb) from the top SNP identified in the Italian Large White breed. Inclusion of the *VRTN* g.20311_20312ins291 mutation in the association analysis carried out in Italian Landrace pigs (allele frequencies of the two alleles were: insertion = 0.67, wild type = 0.33) did not produce any significant result (*P* = 0.088). The level of linkage disequilibrium (*r* ^2^) between the *VRTN* mutation and the top associated SNP identified in Italian Landrace pigs in this region (WU_10.2_7_104470681) was low (*r* ^2^ = 0.05).

In Italian Landrace, we also evaluated in the association analysis the top associated haplotype detected in Italian Large White pigs. No significant association emerged (*P* = 0.38) and different allelic frequencies were detected.

To further compare the genome region in the two breeds, we also analysed the level of linkage disequilibrium between key markers. For example, in our previous study in Italian Large White pigs (Moscatelli et al. 2020), the *VRTN* insertion allele and the top associated marker (MARC0038565) had similar MAF and *β* of association (MAF ~ 0.24 and *β* ~ 0.35) both explained by the high linkage disequilibrium (*r* ^2^ = 0.58). We therefore analysed the linkage disequilibrium between the same two markers in the Italian Landrace population. In this breed linkage disequilibrium was much lower (*r* ^2^ = 0.07). In Italian Landrace pigs we also further evaluated the level of linkage disequilibrium between the *VRTN* marker and the other two significant SNPs (M1GA0010653 and H3GA0022659) detected in this study in the Italian Large White population (Table S4). Again, a poor linkage disequilibrium was evidenced in the Italian Landrace breed (*r* ^2^ = 0.06 and *r* ^2^ = 0.14 respectively).

A comparative haploblock analysis of this region in both Italian Landrace and Italian Large White, considering shared DNA markers between the two datasets, showed some differences in the structure of linkage disequilibrium that could be potentially involved in the contrasting results between the two breeds (Fig. S4). Finally, we also investigated the whole set of haplotypes coming from the related haploblock and again no association was evidenced in the Italian Landrace breed (Table S6).

## Discussion

Among the traits directly or indirectly affecting the reproduction efficiency of the sows, the number of teats in pigs is considered one of the parameters with the highest heritability. For this trait, most studies have indicated medium to high heritability (Rohrer & Nonneman 2017). Genomic heritability estimated in our analyses confirmed what was reported in a previous estimation we carried out in the Italian Large White breed, but with a lower number of pigs (Moscatelli et al. 2020). Our estimated genomic heritability obtained for the two breeds ($h_{G}^{2}$ ranged from 0.25 to 0.43) is also similar to what was described by other authors in different pig breeds and populations using genomic based approaches (Duijvesteijn et al. 2014; Lopes et al. 2014; Arakawa et al. 2015; Balzani et al. 2016; Rohrer & Nonneman 2017; Lee et al. 2019; van Son et al. 2019; Moscatelli et al. 2020). It is interesting to note that in both heavy pig breeds haplotype‐based estimation of $h_{G}^{2}$ resulted in a substantial increase of its value compared to the $h_{G}^{2}$ based on the single‐marker genome analysis ($h_{G}^{2}$ = 0.31 obtained using haplotypes vs $h_{G}^{2}$ = 0.25 obtained using single‐SNPs in Italian Large White and $h_{G}^{2}$ = 0.43 vs. $h_{G}^{2}$ = 0.30 in Italian Landrace). Therefore, a haplotype analysis extracted more information from the dataset and was able to recover a fraction of the so‐called missing heritability, as already demonstrated in several other studies (e.g. Ehret et al. 2012).

It is also interesting to note that results of the GWAS in the two heavy pig breeds did not report any overlapping QTL region for teat number. This is surprising considering that the two breeds may share a quite similar genetic background as already demonstrated by whole‐genome resequencing and genotyping data (Schiavo et al. 2020a,b; Bovo et al. 2020b). However, the two breeds have divergent selection histories that might have shaped their genome and fixed various genomic regions affecting the analysed trait. This consideration could be supported by the characteristics of the QTL regions reported in the two breeds: few QTL regions in the Italian Large White breed (only three significant regions) with quite high MAF, indicating balanced segregation of the alternative alleles in the population captured both using single‐marker and haplotype‐based analysis (with the exception for one QTL region); a larger number of QTL regions in the Italian Landrace breed (12 QTL regions), with low MAF and mainly detected with the haplotype‐based analysis. In the latter breed, it seems that the haplotype approach worked better in capturing low frequency QTL alleles that could not be detected by a single‐marker analysis. Low frequency QTL alleles might be more difficult to be detected using a standard SNP chip, due to the low linkage disequilibrium of the single‐SNPs with causative mutations that do not have an ancestral and common origin across breeds (as a possible result of the ascertainment bias in the selection of the SNPs included in the SNP chip used in the study). It is also clear that alleles (haplotypes) with low MAF could potentially identify false‐positive QTL regions. However, higher estimated genomic heritability of the haplotype analysis in this breed might indicate that most of the haplotype‐detected QTL regions are actually true QTL regions, as we already reported in another study using a similar approach (Bovo et al. 2020a). Moreover, the low MAF at several QTL regions in this breed (captured by the haplotypes; Fig. S1) is also in line to the low frequency of pigs having fewer than 14 teats that we purposely included in the study (6.4%; Table S3). To maximise the variability for this trait, that in the Italian heavy pigs is in some way artificially truncated for the lowest part of the tail distribution by the constant selection against animals with fewer than 14 teats (pigs of these breeds must have at least 14 teats to be registered to their Herd Books), we were able to genotype 124 out of 1941 pigs with a number of teats lower than 14, by selecting animals from the Italian Landrace population that had blood stored in the ANAS biobank (these animals were then eliminated from the Herd Book of the breed).

Other interesting insights emerged by comparing the results of the QTL identified in this study in the two breeds and considering also results of previous studies in the same populations and in other populations. Particularly, in the Italian Large White pigs, we expected to identify a strong QTL signal on SSC7 in the *VRTN* gene region as our previous studies pointed out that variability in this gene might be directly involved in affecting teat numbers (Dall'Olio et al. 2018; Moscatelli et al. 2020). Both single‐marker and haplotype‐based GWAS showed that the most significant QTL region in the Italian Large White breed completely overlapped the *VRTN* gene region, further confirming the candidacy of the variability in this gene. However, no major QTL segregates in this region in the Italian Landrace breed. This is puzzling considering that the *VRTN* g.20311_20312ins291 alleles segregate with a quite balanced frequency also in the Italian Landrace breed, similarly to the results we already reported in a previous study (Fontanesi et al. 2014). However, the inclusion in the association study of the *VRTN* g.20311_20312ins291 mutation confirmed the lack of association between the *VRTN* gene and the number of teats in the Italian Landrace pigs. Moreover, the genome architecture of this region in the Italian Landrace and in the Italian Large White breed seems quite different as it could be inferred from the linkage disequilibrium and haploblock analyses we carried out. Therefore, it seems that there could be breed specific differences on the effect of variability in this SSC7 genome region on the number of teats, as also reported by other authors for the same trait or for the correlated number of vertebrae traits (Rohrer et al. 2015; Zhang et al. 2015, 2016; Park et al. 2017; van Son et al. 2019). Haploblock structure of this SSC7 region between the two breeds was different (Fig. S4). Overall, the two breeds showed a different *P* of association for the haplotypes within the top associated haploblock detected in Italian Large White pigs (Table S6).

Variability in other candidate genes, including the *latent transforming growth factor binding protein 2* (*LTBP2*) and the *ATP binding cassette subfamily D member 4* (*ABCD4*) genes, mapped in this region, has been proposed to explain part of the effects that cannot be explained by the *VRTN* polymorphisms (Zhang et al. 2016; Park et al. 2017; van Son et al. 2019). Considering that we did not identify any QTL signal in this region in the Italian Landrace population, pigs of this breed might be homozygous at these other QTL alleles (that could cover the *VRTN* allele effects) or other breed specific effects could counterbalance the expected effect derived by the *VRTN* gene variants. The lack of QTL reported in this region in the Italian Landrace breed differentiate this heavy pig population from other Landrace populations in which QTL for the number of teats have been reported in this SSC7 position (van Son et al. 2019). Other studies are needed to characterise in more detail this region in which several QTL for other production traits have been also already reported (e.g. Yue et al. 2003).

Among the QTL regions identified in our study, we could mention a few that have been also detected in other pig populations. On SSC10, a QTL emerged in the Italian Large White breed in the *FRMD4A* gene region, already indicated by van Son et al. (2019) to harbour a QTL for the number of teats in another Large White commercial population and in Duroc pigs. *FRMD4A* encodes a FERM domain‐containing protein that regulates epithelial polarity by connecting ADP ribosylation factor 6 (*ARF6*), which is a central player in actin cytoskeleton dynamics and membrane trafficking, with the Par protein complex (Ikenouchi & Umeda 2010).

The most significant signal obtained in the Italian Landrace breed was on SSC15 in a region that was already reported to contain a QTL region for number of teats (including the *ARL4C* gene) in another Landrace population (van Son et al. 2019) further confirming the results we obtained. The closest annotated genes were the *transient receptor potential* *cation* *channel subfamily M member 8* (*TRPM8*) and the *secreted* *phosphoprotein 2* (*SPP2*) genes that based on their role cannot be considered obvious candidates. *TRPM8* encodes a receptor‐activated non‐selective cation channel involved in detection of sensations such as coolness that also plays a role in prostate cancer cell migration whereas SPP2 is involved in retinitis pigmentosa in humans and its function is to respond to elevated platelet cytosolic Ca^2+^.

The obtained results indicated that the Italian Large White and Italian Landrace breeds have a different structure on the segregating QTL affecting teat number. It is worth speculation that this result (if also further extended for other reproductive traits) could contribute to justify, at least in part, the common practice of producing hybrid F1 gilts by crossing Italian Large White and Italian Landrace to exploit heterosis for reproductive related traits that derives by combing different alleles segregating in the two pure breeds.

Both single marker and haplotype‐based genome wide association studies captured QTL regions that were complementary in most cases. In pig populations where the haplotype structure or the level of linkage between QTL alleles and SNPs is not known, it is useful to use both methodologies to fully exploit and dissect the genome of the pigs of different populations.

## Conflict of interest

The authors declare they do not have any competing interests.

## Supporting information

**Figure S1** Quantile‐quantile plots of the genome‐wide association studies (GWAS) carried out in the Italian Large White (ILW) and Italian Landrace (IL) breeds using the single‐marker‐ (SNP) and haplotype‐ (Haplotype) based approaches. Inflation factor (*λ*_GC_) is reported.
**Figure S2** Boxplots showing the allelic effects of the top associated SNPs and haplotype regions for the number of teats in the Italian Large White population. Haplotypes have been treated as bi‐allelic variants (H = haplotype allele and N = other N alleles). In red is highlighted the average number of teats. Genome region SSC7:97435001 bp is also presented in Fig. 3.
**Figure S3** Boxplots showing the allelic effects of the top associated SNPs and haplotype regions for the number of teats in the Italian Landrace population. Haplotypes have been treated as bi‐allelic variants (H = haplotype allele and N = other N alleles). In red is highlighted the average number of teats. Genome region SSC15:134400001 bp is also presented in Fig. 3.
**Figure S4** Pairwise linkage disequilibrium (LD) analysis of the *VRTN* gene region (SSC7) in (A) Italian Large White (ILW) and (B) Italian Landrace (IL) pigs. Only markers shared between the two populations are showed. LD was measured as *R* ^2^ and is presented in each box coloured considering the magnitude of linkage. The associated SNP and haplotypes detected in the ILW pigs are highlighted with a red star symbol whereas position of the *VRTN* gene is marked with a green arrow. DNA markers within the top associated haplotype (CHR7_B973_97200001_97670001_GAGAG) are marked with a red triangle. Frequency of the different haplotypes (MAF > 0.01) are also reported.
**Table S1** Information on the datasets used in the single‐marker (SNP) and haplotype‐based (Haplotype) genome‐wide association studies (GWAS) carried out in Italian Large White and Italian Landrace breeds.
**Table S2** Teat counts in the Italian Large White pig population.
**Table S3** Teat counts in the Italian Landrace pig population.
**Table S4** Genome regions associated with the number of teats in Italian Large White and Italian Landrace pigs. Results are stratified by population, genome scan and chromosome.
**Table S5** Comparison between pig populations in allele frequency, β and *P* of association of the top associated marker identified in the Italian Large White pigs.
**Table S6** Comparison between pig populations in allele frequency, β and *P* of association of the top associated haplotype within the *VRTN* region (and all other haplotypes in the haploblock) detected in the Italian Large White pigs.

## Data Availability

The datasets used and analysed during the current study are available from the corresponding author on reasonable request.
