# Supplementary material for: Single‐marker and haplotype‐based genome‐wide association studies for the number of teats in two heavy pig breeds
Source: Anim Genet. 2021 Jun 6;52(4):440–50. doi: 10.1111/age.13095 (PMC8362157; doi:10.1111/age.13095)

Supplementary material

**Single-marker and haplotype-based genome-wide association studies** **for the number of teats in two heavy pig breeds**

S. Bovo^1,*^, M. Ballan^1,*^, G. Schiavo^1^, A. Ribani^1^, S. Tinarelli^1,2^, V.J. Utzeri^1^, S. Dall’Olio^1^, M. Gallo^2^ and L. Fontanesi^1^

^1^ Department of Agricultural and Food Sciences, Division of Animal Sciences, University of Bologna, Viale Fanin 46, 40127 Bologna, Italy.

^2^ Associazione Nazionale Allevatori Suini (ANAS), Via Nizza 53, 00198 Roma, Italy.

^*^ Equal contribution

**Table S1.** Information on the datasets used in the single-marker (SNP) and haplotype-based (Haplotype) genome-wide association studies (GWAS) carried out in Italian Large White and Italian Landrace breeds.

|  | **Italian Large White** | | **Italian Landrace** | |
| --- | --- | --- | --- | --- |
| **Parameter** | **SNP** | **Haplotype** | **SNP** | **Haplotype** |
| Genotyped animals (*n*) | 3974 | 3974 | 1943 | 1943 |
| Animals used in GWAS (*n*) | 3888 | 3888 | 1941 | 1941 |
| Genotyped DNA markers (*n*) | 36759 | 196927 | 50789 | 237223 |
| Genotyped DNA markers used in GWAS (*n*) | 36243 | 196394 | 50450 | 237087 |
| Genomic heritability $\text{h}_{\text{G}}^{\text{2}}$ (standard error) | 0.25 (0.02) | 0.31 (0.03) | 0.30 (0.03) | 0.43 (0.04) |
| Inflation factors (λ_GC_) | 0.95 | 0.99 | 0.98 | 0.91 |

**Table S2.** Teat counts in the Italian Large White pig population.

| **Teat number class** | **Total no. of pigs (%)^§^** | **No. of gilts (%)** | **No. of sows (%)** | **No. of castrated males (%)** |
| --- | --- | --- | --- | --- |
| 12 | 1 (0.03) | 1 (0.06) | 0 (0) | 0 (0) |
| 13 | 2 (0.05) | 2 (0.12) | 0 (0) | 0 (0) |
| 14 | 1807 (46.48) | 810 (50.56) | 626 (44.08) | 371 (42.84) |
| 15 | 1182 (30.4) | 464 (28.96) | 454 (31.97) | 264 (30.48) |
| 16 | 784 (20.16) | 286 (17.85) | 298 (20.99) | 200 (23.09) |
| 17 | 95 (2.44) | 36 (2.25) | 34 (2.39) | 25 (2.89) |
| 18 | 14 (0.36) | 3 (0.19) | 6 (0.42) | 5 (0.58) |
| 19 | 2 (0.05) | 0 (0) | 2 (0.14) | 0 (0) |
| 20 | 1 (0.03) | 0 (0) | 0 (0) | 1 (0.12) |
| TOTAL | 3888 | 1602 | 1420 | 866 |

^§^ Percentages are given within class.

**Table S3.** Teat counts in the Italian Landrace pig population.

| **Teat number class** | **Total no. of pigs (%)^§^** | **No. of gilts (%)** | **No. of sows (%)** | **No. of castrated males (%)** |
| --- | --- | --- | --- | --- |
| 10 | 124 (6.39) | 0 (0) | 124 (7.88) | 0 (0) |
| 14 | 972 (50.08) | 186 (70.99) | 721 (45.83) | 65 (61.32) |
| 15 | 682 (35.14) | 56 (21.37) | 597 (37.95) | 29 (27.36) |
| 16 | 153 (7.88) | 20 (7.64) | 122 (7.76) | 11 (10.38) |
| 17 | 7 (0.36) | 0 (0) | 6 (0.38) | 1 (0.94) |
| 18 | 3 (0.15) | 0 (0) | 3 (0.19) | 0 (0) |
| TOTAL | 1941 | 262 | 1573 | 106 |

^§^ Percentages are given within class.

**Table S4.** Genome regions associated with the number of teats in Italian Large White and Italian Landrace pigs. Results are stratified by population, genome scan and chromosome.

| **Genome scan^1^** | **SSC^2^** | **Pos^3^** | **Marker^4^** | **Min/Maj^5^** | **MAF^6^** | ***β*^7^** | ***P*^8^** |
| --- | --- | --- | --- | --- | --- | --- | --- |
| **Italian Large White** |  |  |  |  |  |  |  |
| Haplotype-based | 7 | 97435001 | CHR7_B973_97200001_97670001_GAGAG | H/N | 0.426 | 0.188 | 1.61E-15 |
| Haplotype-based | 7 | 97535001 | CHR7_B974_97300001_97770001_GAGGA | H/N | 0.421 | 0.167 | 2.73E-12 |
| Haplotype-based | 7 | 97635001 | CHR7_B975_97400001_97870001_AGGAG | H/N | 0.408 | 0.176 | 2.03E-12 |
| Single-marker | 7 | 97652632 | MARC0038565 | A/G | 0.482 | -0.163 | 2.65E-12 |
| Haplotype-based | 7 | 97735001 | CHR7_B976_97500001_97970001_AGGAGGGA | H/N | 0.387 | 0.153 | 2.39E-09 |
| Single-marker | 7 | 97795647 | M1GA0010653 | A/G | 0.476 | -0.143 | 2.94E-09 |
| Haplotype-based | 7 | 97835001 | CHR7_B977_97600001_98070001_GGAGGGAAAG | H/N | 0.387 | 0.143 | 1.84E-08 |
| Single-marker | 7 | 97881096 | H3GA0022659 | A/G | 0.425 | -0.127 | 2.07E-07 |
| Single-marker | 10 | 47730924 | ALGA0059138 | C/A | 0.474 | -0.121 | 9.67E-07 |
| Single-marker | 10 | 47751164 | M1GA0014145 | A/G | 0.477 | -0.135 | 2.83E-08 |
| Single-marker | 10 | 47805800 | ALGA0103761 | G/A | 0.466 | -0.136 | 3.64E-08 |
| Haplotype-based | 10 | 47935001 | CHR10_B478_47700001_48170001_CCAGAAGGAAA | H/N | 0.283 | -0.134 | 7.82E-08 |
| Haplotype-based | 10 | 48035001 | CHR10_B479_47800001_48270001_GAAGGAAA | H/N | 0.367 | -0.129 | 1.56E-08 |
| Single-marker | 12 | 24723142 | MARC0031045 | G/A | 0.358 | 0.117 | 1.03E-06 |
| **Italian Landrace** |  |  |  | / |  |  |  |
| Haplotype-based | 3 | 117000001 | CHR3_B1169_116800001_117200001_GGGAGACAAAAAGGACCAACAAAG | H/N | 0.016 | -0.926 | 1.38E-07 |
| Haplotype-based | 3 | 120600001 | CHR3_B1205_120400001_120800001_GGCCGAGAGGGAAAAGAA | H/N | 0.014 | -1.048 | 8.40E-08 |
| Haplotype-based | 6 | 113000001 | CHR6_B1129_112800001_113200001_GGGAG | H/N | 0.062 | -0.466 | 1.95E-07 |
| Haplotype-based | 6 | 113100001 | CHR6_B1130_112900001_113300001_GGGAG | H/N | 0.062 | -0.466 | 1.95E-07 |
| Haplotype-based | 8 | 4500001 | CHR8_B44_4300001_4700001_AGGCAAAAGAAGAAG | H/N | 0.011 | -1.142 | 9.30E-09 |
| Haplotype-based | 11 | 18900001 | CHR11_B188_18700001_19100001_GAGAAAGCA | H/N | 0.034 | -0.529 | 1.55E-07 |
| Haplotype-based | 13 | 189100001 | CHR13_B1890_188900001_189300001_AGGAAGGGAA | H/N | 0.016 | -0.957 | 5.43E-09 |
| Haplotype-based | 13 | 189200001 | CHR13_B1891_189000001_189400001_GAAGGGAAAG | H/N | 0.011 | -1.019 | 1.63E-07 |
| Haplotype-based | 13 | 189400001 | CHR13_B1893_189200001_189600001_AAAGGGGAGAAA | H/N | 0.017 | -0.945 | 3.20E-08 |
| Single-marker | 14 | 23582019 | WU_10.2_14_25047530 | A/G | 0.078 | -0.445 | 3.18E-07 |
| Haplotype-based | 14 | 114800001 | CHR14_B1147_114600001_115000001_GAAAAGAG | H/N | 0.051 | -0.547 | 1.79E-07 |
| Haplotype-based | 14 | 132300001 | CHR14_B1322_132100001_132500001_AAGAAGAGGGAAAG | H/N | 0.014 | -0.953 | 1.73E-07 |
| Haplotype-based | 15 | 19300001 | CHR15_B192_19100001_19500001_AGGGAGGGGAAGAA | H/N | 0.013 | -0.877 | 6.29E-08 |
| Haplotype-based | 15 | 19400001 | CHR15_B193_19200001_19600001_AGGGGAAGAAGGA | H/N | 0.014 | -0.851 | 1.11E-07 |
| Haplotype-based | 15 | 128600001 | CHR15_B1285_128400001_128800001_ACAAGGAAAGAGGAAAAGG | H/N | 0.063 | -0.490 | 1.79E-07 |
| Haplotype-based | 15 | 128700001 | CHR15_B1286_128500001_128900001_AAGGAAAGAGGAAAAGGAACGG | H/N | 0.055 | -0.559 | 2.58E-08 |
| Haplotype-based | 15 | 129600001 | CHR15_B1295_129400001_129800001_GCAGAGAAAAGCGGGGG | H/N | 0.036 | -0.768 | 1.81E-09 |
| Haplotype-based | 15 | 129700001 | CHR15_B1296_129500001_129900001_GAAAAGCGGGGGGAAC | H/N | 0.035 | -0.808 | 2.98E-10 |
| Haplotype-based | 15 | 129800001 | CHR15_B1297_129600001_130000001_AGCGGGGGGAACAAAA | H/N | 0.035 | -0.746 | 7.97E-09 |
| Haplotype-based | 15 | 129900001 | CHR15_B1298_129700001_130100001_CGGGGGGAACAAAAAGGA | H/N | 0.034 | -0.835 | 1.24E-10 |
| Haplotype-based | 15 | 134200001 | CHR15_B1341_134000001_134400001_AGAGGGACGAAAAAAGAA | H/N | 0.034 | -0.951 | 1.84E-13 |
| Haplotype-based | 15 | 134300001 | CHR15_B1342_134100001_134500001_GGACGAAAAAAGAAGAA | H/N | 0.035 | -0.868 | 1.72E-11 |
| Haplotype-based | 15 | 134400001 | CHR15_B1343_134200001_134600001_GAAAAAAGAAGAAGGG | H/N | 0.030 | -1.062 | 8.08E-16 |
| Haplotype-based | 15 | 135700001 | CHR15_B1356_135500001_135900001_GAAAAAGGGGAACGA | H/N | 0.074 | -0.454 | 2.08E-07 |
| Haplotype-based | 15 | 136400001 | CHR15_B1363_136200001_136600001_GGGGGAAGAGC | H/N | 0.055 | -0.570 | 6.78E-10 |
| Haplotype-based | 15 | 136900001 | CHR15_B1368_136700001_137100001_AGGAGGAGAGGGCGA | H/N | 0.055 | -0.550 | 1.68E-08 |
| Haplotype-based | 15 | 137000001 | CHR15_B1369_136800001_137200001_AGGAGAGGGCGAGAAGC | H/N | 0.060 | -0.638 | 4.51E-11 |
| Haplotype-based | 16 | 57400001 | CHR16_B573_57200001_57600001_GAGAA | H/N | 0.024 | -0.806 | 6.15E-08 |
| Haplotype-based | 16 | 68700001 | CHR16_B686_68500001_68900001_GAACGAAGAAGGGGA | H/N | 0.017 | -0.993 | 5.08E-09 |

^1^ Genome scans performed within each pig population (Italian Large White, ILW; Italian Landrace, IL). SNPs and Haplotypes indicates which DNA markers have been used to carry out the genome scans.

^2^ *Sus scrofa* chromosome.

3 Position, in basepairs, on the *Sus scrofa* reference genome (v.11.1).

^4^ DNA marker identifier reported in the chip panels. For haplotypes, the haploblock identifier (chromosome specific) is reported.

5 Minor/Major alleles. Haplotypes have been treated as bi-allelic variants (H = haplotype allele and N = other *N* alleles).

6 Minor allele frequency.

^7^ Regression coefficient. A positive value indicates that the no. of teats increases with the increasing of the number of copies of the minor allele. A negative value indicates that the no. of teats decreases with the increasing of the number of copies of the minor allele.

8 *P* at the Wald test (GEMMA).

**Table S5.** Comparison between pig populations in allele frequency, ***β*** and *P* of association of the top associated marker identified in the Italian Large White pigs.

|  |  |  | **Italian Large White** | | | | **Italian Landrace** | | | |
| --- | --- | --- | --- | --- | --- | --- | --- | --- | --- | --- |
| **DNA Marker** | **Position^1^** | **Candidate gene** | **Min/Maj^2^** | **MAF^3^** | ***β*^4^** | ***P*^5^** | **Min/Maj** | **MAF** | ***β*** | ***P*** |
| MARC0038565 | 7: 97652632 | *VRTN* | A/G | 0.482 | -0.16 | 2.65E-12 | A/G | 0.091 | -0.08 | 0.37 |
| M1GA0014145 | 10: 47751164 | *FERMD4A* | A/G | 0.477 | -0.13 | 2.83E-08 | A/G | 0.387 | -0.05 | 0.29 |
| MARC0031045 | 12: 24723142 | *HOXB1* | G/A | 0.358 | 0.11 | 1.03E-06 | G/A | 0.457 | 0.05 | 0.74 |

^1^ *Sus scrofa* chromosome and position in basepairs. Reference genome refers to version 11.1

2 Minor/Major alleles. Haplotypes have been treated as bi-allelic variants (H = haplotype allele and N = other *N* alleles).

3 Minor allele frequency.

^4^ Regression coefficient. A positive value indicates that the no. of teats increases with the increasing of the number of copies of the minor allele. A negative value indicates that the no. of teats decreases with the increasing of the number of copies of the minor allele.

5 *P* at the Wald test (GEMMA).

**Table S5.** Comparison between pig populations in allele frequency, *β* and *P* of association of the top associated haplotype (CHR7_B973_97200001_97670001_GAGAG) within the *VRTN* region (and all other haplotypes in the haploblock) detected in the Italian Large White pigs.

|  | **Italian Large White** | | | | **Italian Landrace** | | | |
| --- | --- | --- | --- | --- | --- | --- | --- | --- |
| Haplotype (H)* | **Min/Maj^1^** | **MAF^2^** | ***β*^3^** | ***P*^4^** | **Min/Maj^2^** | **MAF^3^** | ***β*^4^** | ***P*^5^** |
| GAGAG | H/N | 0.426 | 0.19 | 1.61E-15 | N/H | 0.312 | -0.05 | 0.38 |
| GAAAA | H/N | 0.314 | -0.1 | 2.19E-05 | H/N | 0.036 | -0.03 | 0.81 |
| AAGAA | H/N | 0.106 | -0.12 | 6.28E-04 | H/N | 0.003 | -0.12 | 0.74 |
| AGGCG | H/N | 0.021 | -0.1 | 0.16 | H/N | 0.001 | 0.61 | 0.32 |
| AGGAA | H/N | 0.039 | 0.07 | 0.19 | * | * | * | * |
| GAGAA | H/N | 0.021 | -0.08 | 0.20 | H/N | 0.001 | 0.43 | 0.49 |
| AAGAG | H/N | 0.03 | -0.06 | 0.31 | * | * | * | * |
| GAAAG | H/N | 0.021 | -0.03 | 0.68 | H/N | 0.147 | -0.03 | 0.64 |
| AAGCG | H/N | 0.01 | 0.03 | 0.73 | * | * | * | * |
| GGACA | * | * | * | * | H/N | 0.01 | 0.04 | 0.86 |
| GGGCA | * | * | * | * | H/N | 0.018 | -0.16 | 0.33 |
| AAGCA | * | * | * | * | H/N | 0.020 | -0.18 | 0.38 |

1 Minor/Major alleles. Haplotypes have been treated as bi-allelic variants (H = haplotype allele and N = other *N* alleles).

2 Minor allele frequency.

^3^ Regression coefficient. A positive value indicates that the no. of teats increases with the increasing of the number of copies of the minor allele. A negative value indicates that the no. of teats decreases with the increasing of the number of copies of the minor allele.

4 *P* at the Wald test (GEMMA).

* The star symbol identifies haplotypes not detected in the population or having a MAF < 0.01.

**Figure S1.** Quantile-quantile plots of the genome-wide association studies (GWAS) carried out in the Italian Large White (ILW) and Italian Landrace (IL) breeds using the single-marker- (SNP) and haplotype- (Haplotype) based approaches. Inflation factor (λ_GC_) is reported.


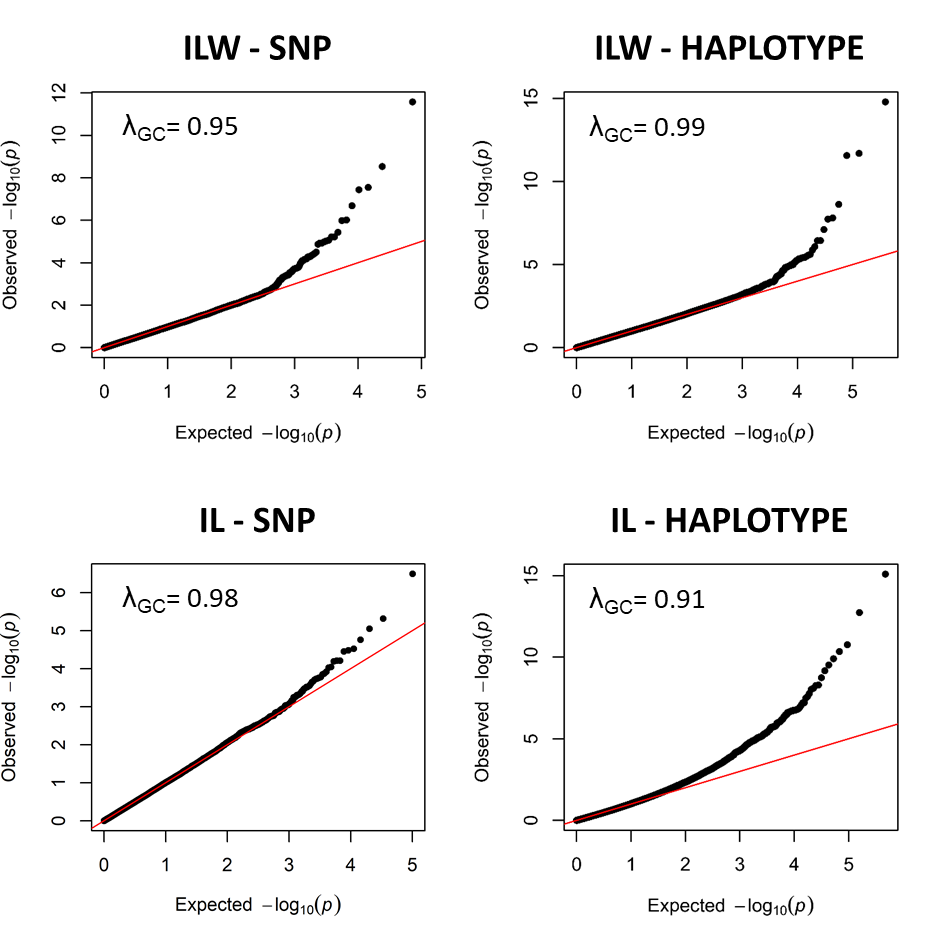


**Figure S2.** Boxplots showing the allelic effects of the top associated SNPs and haplotype regions for the number of teats in the Italian Large White population. Haplotypes have been treated as bi-allelic variants (H = haplotype allele and N = other *N* alleles). In red is highlighted the average number of teats. Genome region SSC7:97435001 bp is also presented in Fig. 3.


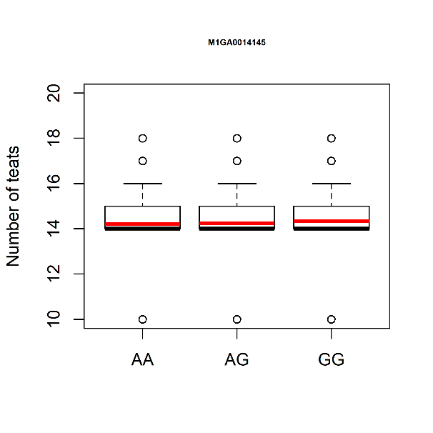

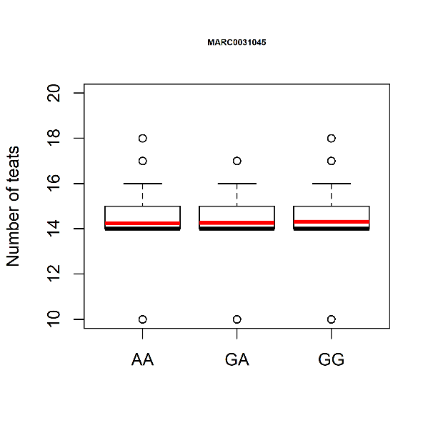

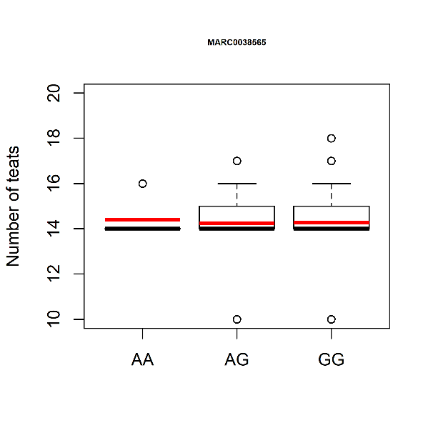

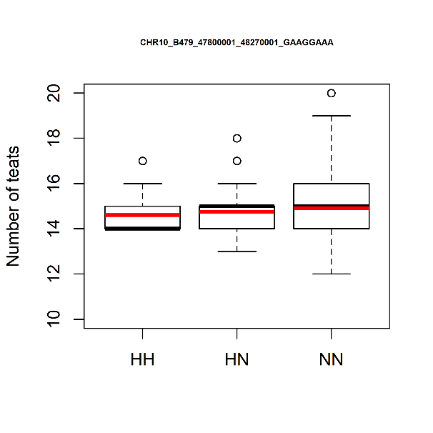

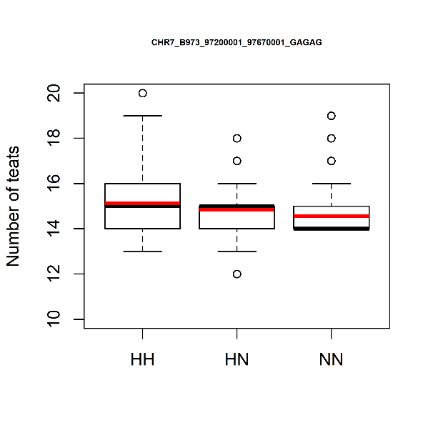


**Figure S3.** Boxplots showing the allelic effects of the top associated SNPs and haplotype regions for the number of teats in the Italian Landrace population. Haplotypes have been treated as bi-allelic variants (H = haplotype allele and N = other *N* alleles). In red is highlighted the average number of teats. Genome region SSC15:134400001 bp is also presented in Fig. 3.


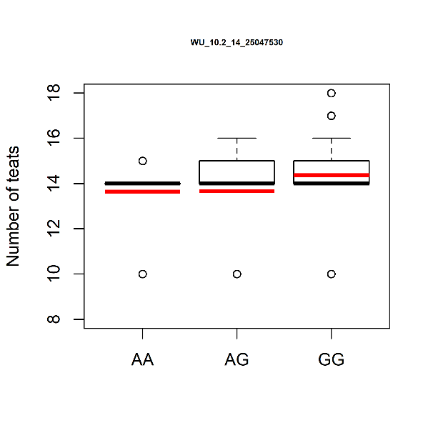

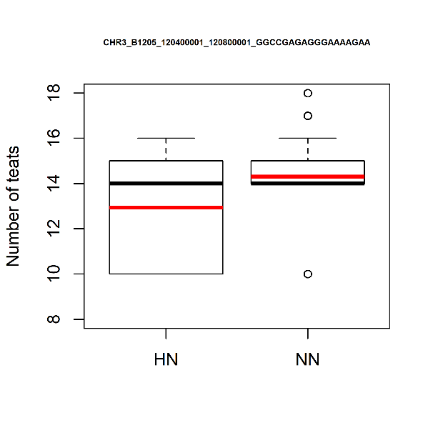

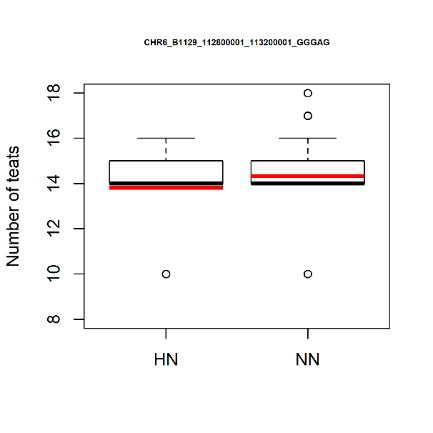

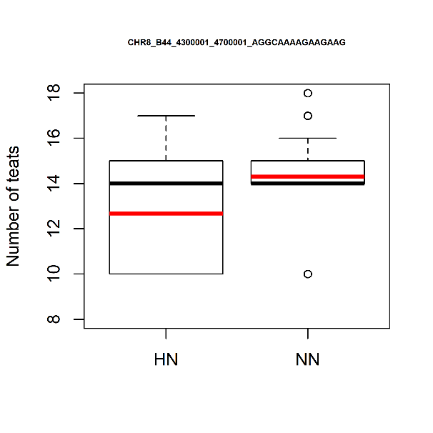

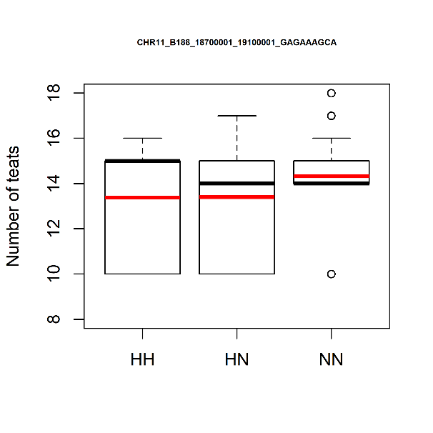

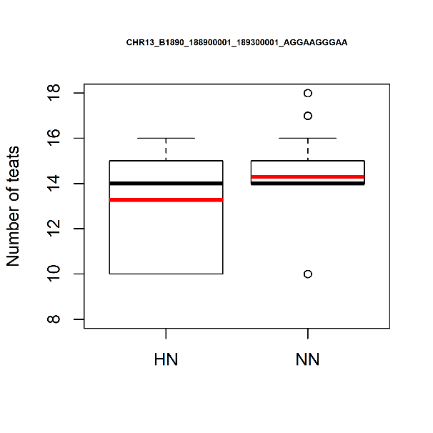


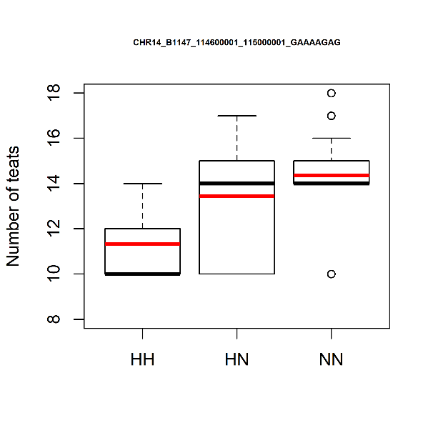

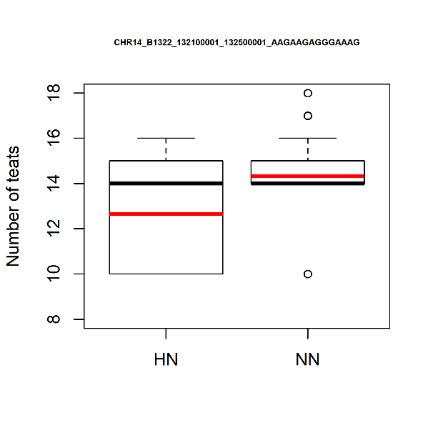

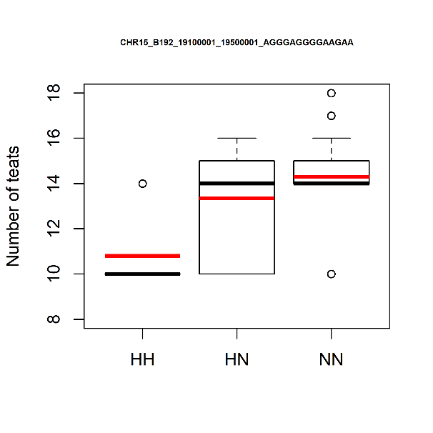

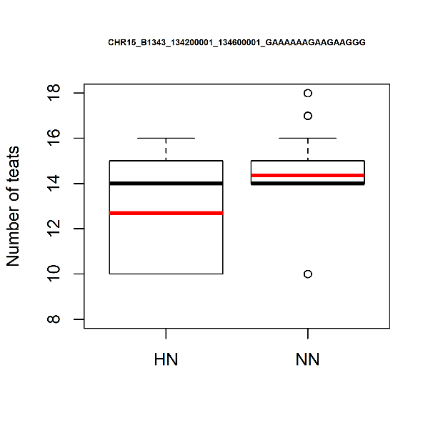

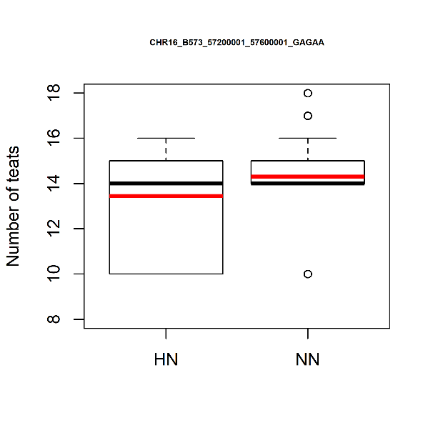

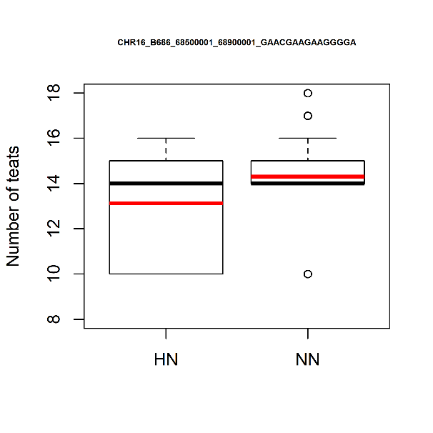


**Figure S4.** Pairwise linkage disequilibrium (LD) analysis of the *VRTN* gene region (SSC7) in A) Italian Large White (ILW) and B) Italian Landrace (IL) pigs. Only markers shared between the two populations are showed. LD was measured as r^2^ and it is presented in each box coloured considering the magnitude of linkage. The associated SNP and haplotypes detected in the ILW pigs are highlighted with a red star symbol whereas position of the *VRTN* gene is marked with a green arrow. DNA markers within the top associated haplotype (CHR7_B973_97200001_97670001_GAGAG) are marked with a red triangle. Ferquency of the different haplotypes (MAF > 0.01) are also reported.


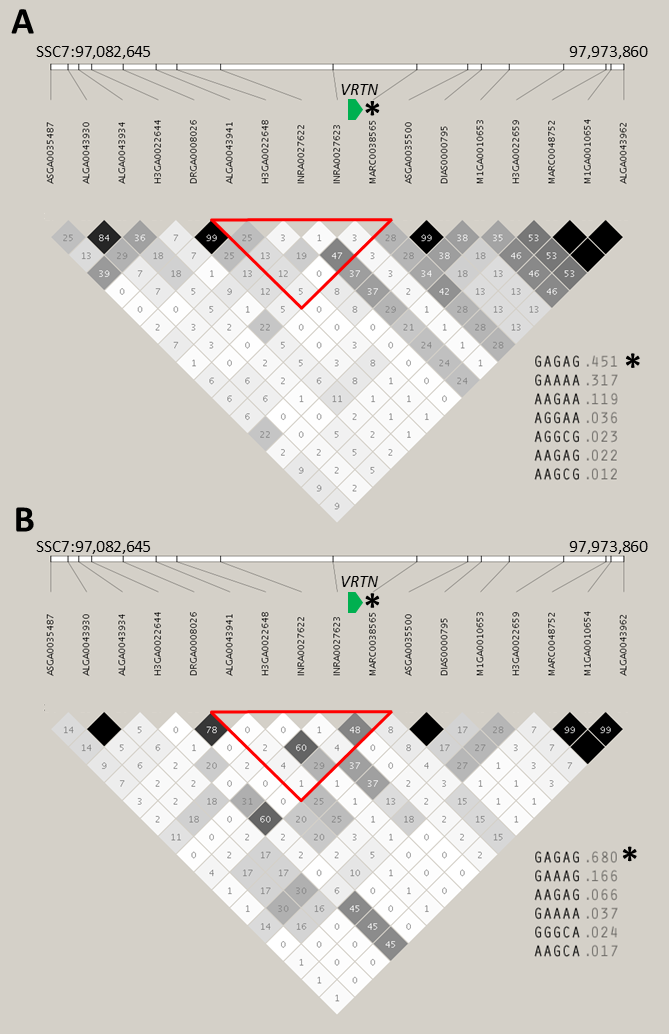

Supplement: Supplementary file 1 — Figure S1 Quantile‐quantile plots of the genome‐wide association studies (GWAS) carried out in the Italian Large White (ILW) and Italian Landrace (IL) breeds using the single‐marker‐ (SNP) and haplotype‐ (Haplotype) based approaches. Inflation factor (λ GC) is reported. Figure S2 Boxplots showing the allelic effects of the top associated SNPs and haplotype regions for the number of teats in the Italian Large White population. Haplotypes have been treated as bi‐allelic variants (H = haplotype allele and N = other N alleles). In red is highlighted the average number of teats. Genome region SSC7:97435001 bp is also presented in Fig. 3. Figure S3 Boxplots showing the allelic effects of the top associated SNPs and haplotype regions for the number of teats in the Italian Landrace population. Haplotypes have been treated as bi‐allelic variants (H = haplotype allele and N = other N alleles). In red is highlighted the average number of teats. Genome region SSC15:134400001 bp is also presented in Fig. 3. Figure S4 Pairwise linkage disequilibrium (LD) analysis of the VRTN gene region (SSC7) in (A) Italian Large White (ILW) and (B) Italian Landrace (IL) pigs. Only markers shared between the two populations are showed. LD was measured as R 2 and is presented in each box coloured considering the magnitude of linkage. The associated SNP and haplotypes detected in the ILW pigs are highlighted with a red star symbol whereas position of the VRTN gene is marked with a green arrow. DNA markers within the top associated haplotype (CHR7_B973_97200001_97670001_GAGAG) are marked with a red triangle. Frequency of the different haplotypes (MAF > 0.01) are also reported. Table S1 Information on the datasets used in the single‐marker (SNP) and haplotype‐based (Haplotype) genome‐wide association studies (GWAS) carried out in Italian Large White and Italian Landrace breeds. Table S2 Teat counts in the Italian Large White pig population. Table S3 Teat counts in the Italian Landrace pig population [file AGE-52-440-s001.docx]
